# Acupoint Catgut-Embedding Therapy Inhibits NF-*κ*B/COX-2 Pathway in an Ovalbumin-Induced Mouse Model of Allergic Asthma

**DOI:** 10.1155/2022/1764104

**Published:** 2022-03-02

**Authors:** Fangzhou Teng, Xuedong Ma, Jie Cui, Xueyi Zhu, Weifeng Tang, Wenqian Wang, Tulake Wuniqiemu, Jingjing Qin, La Yi, Yuting Zong, Chengyong Liu, Shiyuan Wang

**Affiliations:** ^1^Department of Integrative Medicine, Huashan Hospital, Fudan University, Shanghai 200040, China; ^2^Institutes of Integrative Medicine, Fudan University, Shanghai 200040, China; ^3^Gumei Community Health Center of Minhang District of Shanghai, Shanghai 201102, China; ^4^Jiangsu Province Hospital of Chinese Medicine, Affiliated Hospital of Nanjing University of Chinese Medicine, Nanjing 210029, China

## Abstract

Allergic asthma is associated with T helper (Th) 2 cell-biased immune responses and characterized by the airway hyperresponsiveness (AHR). Studies have shown that the acupoint catgut-embedding therapy (ACE) has a therapeutic effect on allergic asthma. However, the relevant mechanism is poorly understood. In present study, female BALB/c mice were sensitized and challenged with ovalbumin (OVA) to establish a model of allergic asthma. AHR was evaluated by using airway resistance (*R*_*L*_) and lung dynamic compliance (Cdyn). Airway inflammation and mucus hypersecretion were observed by HE and PAS staining. Inflammatory cells were counted, and related cytokines in bronchoalveolar lavage fluid (BALF) were detected by enzyme-linked immunosorbent assay (ELISA). Pulmonary group 2 innate lymphoid cell (ILC2s) proportions were analyzed by flow cytometry. The expression of nuclear factor *κ*B (NF-*κ*B) and cyclooxygenase-2 (COX-2) was detected by immunostaining. Our results showed that OVA induction resulted in a significant increase in *R*_*L*_, accompanied by a significant decrease in Cdyn. The levels of interleukin- (IL-) 4, IL-13, OVA-specific IgE in BALF, and the percentage of ILC2 in the lungs were markedly increased accompanied by a significant decreased in interferon-*γ* (IFN-*γ*). Furthermore, the expressions of p-NF-*κ*B p65 and COX-2 in airways were significantly upregulated. After ACE treatment, the indicators above were significantly reversed. In conclusion, ACE treatment inhibited the secretion of Th2 cytokines and the proliferation of ILC2s in the lungs, thereby dampening the inflammatory activity in allergic asthma. The underlying mechanism might be related to the inhibition of NF-*κ*B/COX-2 pathway.

## 1. Introduction

Allergic asthma is a complex immunologic and chronic inflammatory disease with a series of symptoms such as wheezing, shortness of breath, chest tightness, and coughing [1], which is closely associated with airway inflammation and airway remodeling, and characterized by the airway hyperresponsiveness (AHR). According to the 2015 Global Burden of Disease Report, asthma is the most common chronic respiratory disease, with an estimated prevalence of 358 million cases, and based on current data, the World Health Organization predicts that the number of patients suffer from asthma will increase by 100 million in 2025 [2].

It is generally accepted that allergic asthma is related to T helper (Th) 2 cell-biased immune responses. The Th2-related cytokines such as IL-4 and IL-13 are of vital importance in the pathological process of asthma by inducing IgE production [3, 4] and airway mucus hypersecretion [5]. Group 2 innate lymphoid cells (ILC2s) are new players in allergic asthma with a powerful ability to produce type 2 cytokines [6]. Studies have verified that, similar to Th2 cells, ILC2s can preferentially produce cytokines such as IL-4 and IL-13 after being stimulated [7], which enables ILC2s to trigger an allergic immune response without the help of Th2 cells [8]. On the other hand, cytokines secreted by Th2 cells such as IL-4 and IL-13 also promote the proliferation and expansion of the ILC2s population in the lungs [9]. Therefore, both Th2 cytokines and ILC2s are responsible for the asthmatic airway inflammation and may interact with each other to enhance the proinflammatory effects. In addition, studies have shown that IL-4 can modify the activation of nuclear factor *κ*B (NF-*κ*B) induced by other agents [10], and NF-*κ*B also plays an important role in the pathogenesis of IL-13-induced tissue damage in asthma [11, 12]. The activation of NF-*κ*B is crucial in the inflammatory response by inducing the transcription of proinflammatory genes and is correlated with airway inflammatory diseases such as asthma [13]. As a downstream of NF-*κ*B, COX-2 has been confirmed to be related to asthma airway inflammation, it can be highly induced by a variety of stimulus such as cytokines [14] and oxidative stress [15], and its expression has also been shown to be significantly elevated in both asthmatic patients and mice compared with normals [16, 17]. The increased COX-2 expression could induce the elevation of prostaglandin *E*_2_ (PGE_2_, the main product of COX-2 induction), leading to the contraction of airway smooth muscle [14]. Studies have shown that COX-2 induced by NF-*κ*B is an important mediator in the lung inflammation, and the expression of COX-2 can also be enhanced by the activation of NF-*κ*B. On the other hand, COX-2 itself also participates in the activation process of NF-*κ*B, leading to an induction of other inflammatory mediators and cells [18].

Acupoint catgut embedding (ACE) is an important component of acupuncture. Based on the theory of meridians and acupoints, ACE implants catgut or other absorbable thread into corresponding acupoints with special needles to produce continuous stimulation. Compared with traditional acupuncture, ACE not only make the daily acupuncture treatment more convenient for patients but also is more adaptable to the modern fast-paced life. Previous study have shown that ACE can effectively improve lung function and ameliorate the symptoms of allergic asthma [19]. However, the mechanism underlining the therapeutic effect of ACE on asthma is still not clear. In present study, the effects of ACE on Th2 inflammatory responses and NF-*κ*B/COX-2 signaling pathway were evaluated to explore its potential mechanism in asthma treatment.

## 2. Methods

### 2.1. Animals

BALB/c mice (female, 6 weeks) were purchased from Shanghai JieSiJie Laboratory Animals Co., LTD. Mice were kept in a specific pathogen free (SPF) environment (temperature 20–24°C,humidity 45%–55%) with a 12 h dark-light cycle and were fed with food/water freely. This study was approved by the Animal Experiment Ethics Committee of Fudan University (ref: 2019-07-HSYY-WY-02). All animal experiments conducted in present study are in compliance with the “Guidelines for the Care and Use of Laboratory Animals.”

### 2.2. Asthma Model Establishment

Forty mice were randomly divided into the control group (NC), the control + acupoint catgut-embedding group (NA), the asthma group (AST), the asthma + acupoint catgut-embedding group (ASA), and the asthma + sham-acupoint catgut-embedding group (ASS), *n* = 8 in each group. To establish a model of allergic asthma, mice were sensitized and challenged with ovalbumin (OVA) by intraperitoneal injection and atomization inhalation as mentioned previously [20]. In brief, on day 0 and day 7 of model building (sensitization stage), mice in groups AST, ASS, and ASA were given a mixture of OVA (20 *μ*g, Grade V; Sigma-Aldrich) and aluminum hydroxide (2 mg, Thermo Scientific) by intraperitoneally injection. From day 14 to 16 (challenge stage), mice were given an inhalation of 3% OVA solution (w/v) for 30 min through an ultrasonic nebulizer (402AI, Yuyue Medical Equipment Co., Ltd., Jiangsu, China). Then, from day 21 to 48, mice were challenged daily. Additionally, mice in the NC and NA groups were sensitized and challenged with saline.

### 2.3. Acupoint Catgut-Embedding Treatment

On day 21, mice in NA and ASA groups received a one-time ACE treatment after nebulization. A disposable catgut embedding needle (diameter 0.7 mm; Gaoguan Medical, Zhenjiang, China) and absorbable catgut (cut into 0.5 cm length, collagen wire, 2-0, 2 cm × 10, BD150101, Boda Co., Ltd., Shandong, China) were used. After mice were anesthetized, ACE needle was performed at a 45° angle towards the spine, with a penetration depth of 0.3 cm, and then the catgut was implanted into Dazhui (DU14), Fengmen (BL12, bilateral), and Feishu (BL13, bilateral) of each mouse. In addition, the mice in the ASS group received sham-acupoint catgut-embedding treatment with the same operation method as the NA and ASA groups, but no catgut was left [21].

### 2.4. Measurement of Airway Hyperresponsiveness

After the last OVA challenge, the AHR of mice in each group was measured and recorded using lung function device (Buxco150 Electronics, Troy, NY, USA) within 24 h. Mice were anesthetized by intraperitoneal injection with sodium pentobarbital (50 mg/kg). After anesthetizing, a tracheotomy was performed, and a tracheal tube was inserted. Mice were placed in a plethysmograph chamber connected with a ventilator by an inserted tracheal tube, exposed to an increasing dose of 0, 3.125, 6.25, and 12.5 mg/mL methacholine (Mch, Sigma-Aldrich) in sequence. Lung dynamic compliance (Cdyn) and airway resistance (*R*_*L*_) were recorded.

### 2.5. Inflammatory Cell Count and Cytokine Detection

After AHR measurement, bronchoalveolarlavage fluid (BALF) of mice in each group was collected. After centrifuged at 500 g for 10 min, the supernatant of BALF was stored at –80°C for cytokine detection. A portion of the remaining cell pellets was resuspended in PBS, and then the cells were classified and counted by a special counter (BC-5000 vet, Mindray, China). ELISA (Cayman Chemical, Michigan, USA) was used to detect the levels of IL-4, IL-13, and OVA-specific IgE in BALF, and the procedures were operated according to the manufacturers' instructions [22].

### 2.6. Histological Studies

The hematoxylin-eosin (HE) staining and periodic acid Schiff (PAS) staining were used to evaluate airway inflammation and mucus secretion, respectively. For HE staining, according to the infiltration of inflammatory cells around the airway, a scoring system was used to assess the degree of inflammation [22]:0, no inflammatory cell was observed; 1, only a few inflammatory cells; 2, airway or vessels were surrounded by inflammatory cells (1-2 cell layers); 3, airway or vessels were surrounded by inflammatory cells (3-5 cell layers); and 4, airway or vessels were surrounded by inflammatory cells (>5 cell layers). For PAS staining, the percentage of positive staining in the area of interest (AOI) was calculated by ImageJ software.

### 2.7. Flow Cytometry Analysis

Lung dissociation kit (130-095-927, Miltenyi Biotec Technology & Trading Co., Ltd.) was used to prepare lung tissues single cell suspension. For ILC2 identification, cells were stained with Fixable Viability Dye eFluor780 (65-0865-18, thermo) and other antibodies for 20 min on ice. Antibodies were used as follows: anti-mouse CD45 eF506 (69-0451-82, thermo), anti-mouse CD90.2 FITC (140304, BioLegend), anti-mouse CD127 PE-Cy7 (135013, Biolegend), anti-mouse conjugated anti-killer cell lectin-like receptor G1 APC (KLRG1; 17-5893-81, thermo), and anti-mouse Lin eF450 (88-7772-72, Thermo). ILC2s were identified as lineage-CD45 + CD127 + CD90.2 + KLRG1+. LSRFortessa flow cytometers (BD Biosciences, San Jose, CA, USA) were used to detected samples, and Flow Jo software version 10.0 was used to analyze the data.

### 2.8. Immunofluorescence and Immunohistochemistry

As mentioned in our previous study [22], briefly, sodium citrate buffer (0.01 M) was used for antigen repair. 3% hydrogen peroxide was used to inactivate endogenous enzymes (immunohistochemistry only). 5% BSA was used for antigen blocking. A fluorescence microscope (Nikon, Eclipse C1) was used to observe immunofluorescence slides, and a conventional microscope (CIC, XSP-C204) was used to observe immunohistochemistry slides. For each slice, three fields of view were randomly selected to take pictures. Antibodies: p-p65 (AF2006, Dilution: 1 : 200, Affinity), COX-2 (ab15191, Dilution: 1 : 100, Abcam), FITC-conjugated goat anti-rabbit IgG (#GB22303, Dilution:1 : 400,Servicebio), and HRP-conjugated goat anti-rabbit IgG (#GB23303, Dilution:1 : 200, Servicebio). Additionally, for immunohistochemistry pictures, area of interest (AOI) was selected, and IPP 6.0 software was used to calculate the integrated option density (IOD); then, the mean fluorescent intensity (MFI, integrated density/area) and average optical density (AOD, IOD/AOI) were used for semiquantitative analysis.

### 2.9. Statistical Analysis

SPSS20.0 and Graphpad 8.0 were used for statistical analysis and graphing. For data that met the normal distribution, one-way ANOVA was used for pairwise comparison. Kruskal-Wallis *H* test was used for pairwise comparison of data that did not conform to the normal distribution. *P* < 0.05 was considered as statistically significant.

## 3. Results

### 3.1. Acupoint Catgut-Embedding Alleviated AHR in OVA-Induced Asthmatic Mice

**Figure 1:**
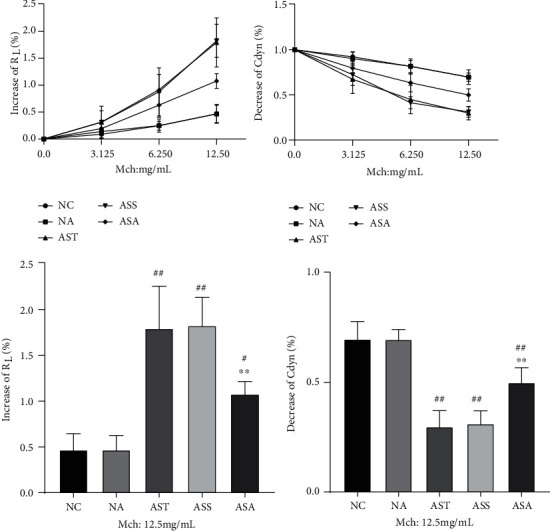
Effect of acupoint catgut embedding on AHR in OVA-induced asthmatic mice. Data are presented as mean ± SD, *n* = 5. ^#^*P* < 0.05, ^##^*P* < 0.01 vs. NC and NA group; ^∗∗^*P* < 0.01 vs. AST and ASS group. NC: control group; NA: control + acupoint catgut-embedding group; AST: asthma group; ASS: asthma + sham-acupoint catgut-embedding group; ASA: asthma + acupoint catgut-embedding group; *R*_*L*_: airway resistance; Cdyn: lung dynamic compliance; Mch: methacholine.

Allergic asthma is characterized by AHR, which is evidenced by airway resistance^increase^ (*R*_*L*_) and lung dynamic compliance^decrease^ (Cdyn) [23]. In present study, we observed these two indicators in each group, and the results (Figure 1) showed that with the increase of methacholine (Mch) concentrations, the *R*_*L*_ (Mch = 12.5 mg/mL, *P*_AST,ASS_ < 0.01, *P*_ASA_ < 0.05) of mice in the AST, ASS, and ASA groups was significantly increased, accompanied by a significant decrease in Cdyn (Mch = 12.5 mg/mL, *P*_AST,ASS,ASA_ < 0.01) compared with the NC and NA groups, indicating the success establishment of the asthma model. In addition, there was no statistical difference in *R*_*L*_ and Cdyn between the AST and ASS groups at 12.5 mg/mL of Mch, as well as between the NC and NA groups. After ACE treatment, both the *R*_*L*_ (Mch = 12.5 mg/mL, *P*_ASA_ < 0.01) and Cdyn (Mch = 12.5 mg/mL, *P*_ASA_ < 0.01) in the ASA group were significantly reversed compared with the AST and ASS groups.

### 3.2. Acupoint Catgut-Embedding Downregulated the Number of Inflammatory Cells in the BALF of Asthmatic Mice

**Figure 2:**
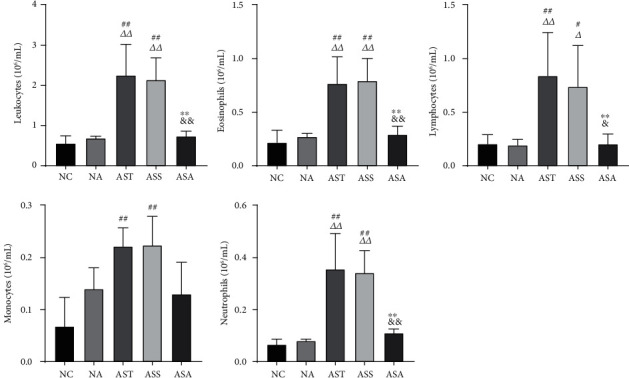
The inflammatory cell count in BALF of each group. Data are presented as mean ± SD, *n* = 5. ^#^*P* < 0.05, ^##^*P* < 0.01 vs. NC group; *^Δ^P* < 0.05, *^ΔΔ^P* < 0.01 vs. NA group; ^∗∗^*P* < 0.01 vs. AST group; ^&^*P* < 0.05, ^&&^*P* < 0.01 vs. ASS group. NC: control group; NA: control + acupoint catgut-embedding group; AST: asthma group; ASS: asthma + sham-acupoint catgut-embedding group; ASA: asthma + acupoint catgut-embedding group.

Next, we classified and counted the inflammatory cells in the BALF of each group of mice. As shown in Figure 2, there was no statistical difference in cell counts between the NC and NA groups, as well as between the AST and ASS groups. Compared with the NC and NA groups, the count of leukocytes (Leu, *P*_AST,ASS_ < 0.01), eosinophils (Eos, *P*_AST,ASS_ < 0.01), lymphocytes (Lym, *P*_AST_ < 0.01, *P*_ASS_ < 0.05), monocytes (Mon, *P*_AST,ASS_ < 0.01), and neutrophils (Neu, *P*_AST,ASS_ < 0.01) was significantly increased in the AST and ASS groups. Compared with the AST and ASS groups, the count of Leu, Eos, Lym, and Neu in the ASA group was significantly decreased, while the count of Mon was not significantly different, but there was a trend of downregulation.

### 3.3. Acupoint Catgut-Embedding Ameliorated Airway Inflammation and Mucus Secretion in OVA-Induced Asthmatic Mice

**Figure 3:**
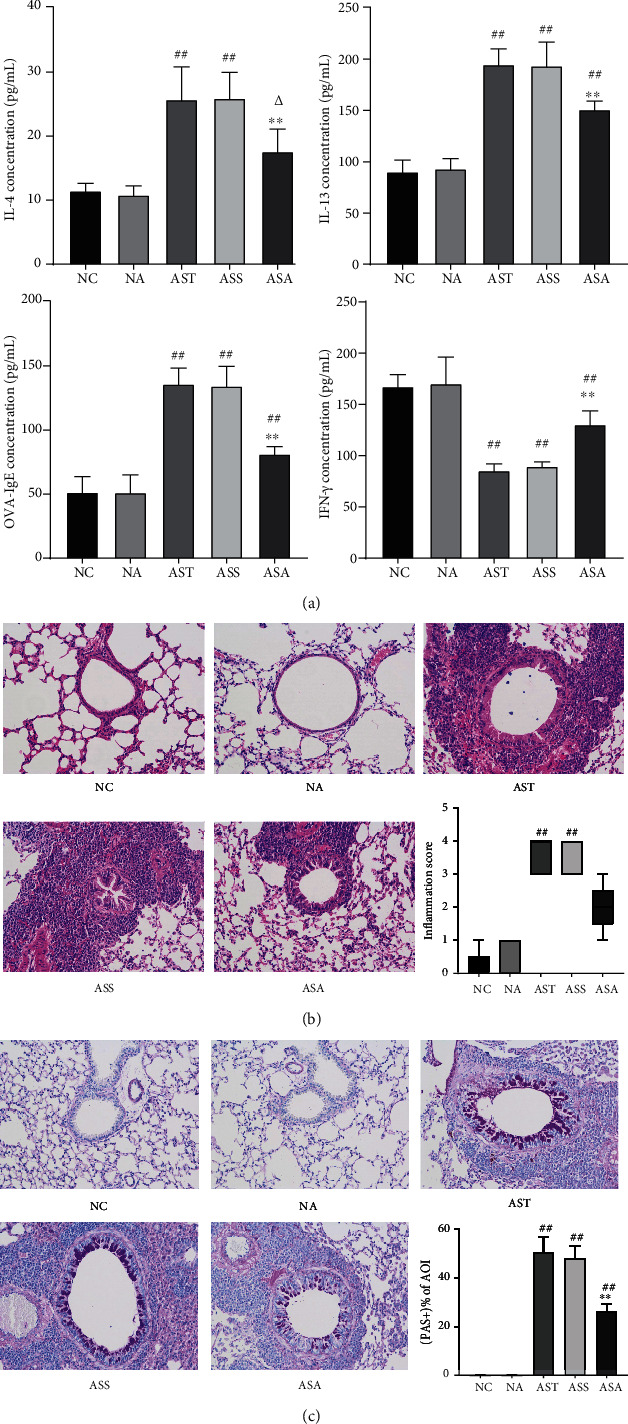
Acupoint catgut-embedding treatment inhibited lung inflammation and mucus hypersecretion in OVA-induced asthmatic mice. (a) Inflammatory cytokines in BALF. (b) Hematoxylin and eosin staining in each group. (c) Periodic acid Schiff staining in each group. Data are presented as mean ± SD/median (P25, P75), *n* = 5. ^##^*P* < 0.01 vs. NC and NA group; *^Δ^P* < 0.05 vs. NA group; ^∗∗^*P* < 0.01 vs. AST and ASS group. NC: control group; NA: control + acupoint catgut-embedding group; AST: asthma group; ASS: asthma + sham-acupoint catgut-embedding group; ASA: asthma + acupoint catgut-embedding group.

IL-4 and IL-13 are main signature cytokines in type 2 immune response and are responsible for asthma and many other allergic inflammatory diseases [24]. We detected the expression levels of IL-4 and IL-13 in the BALF of each group. As shown in Figure 3(a), there was no statistical difference in the expressions of IL-4 and IL-13 between the NC and NA groups, as well as between the AST and ASS groups. Compared with the NC and NA groups, both IL-4 and IL-13 (*P*_AST,ASS_ < 0.01) were significantly elevated in the AST and ASS groups. After ACE treatment, the levels of IL-4 and IL-13 (*P*_AST,ASS_ < 0.01) in the ASA group were significantly reduced compared with the AST and ASS groups. Since IL-4 modulates the synthesis of IgE [25] and OVA was used to induce asthma, we also detected the OVA-specific IgE in BALF. The results showed that there was no statistical difference in OVA-specific IgE level between the NC and NA groups. Compared with the NC and NA groups, the level of OVA-specific IgE was significantly elevated in the AST, ASS, and ASA groups (*P* < 0.01). There was no statistical difference in OVA-specific IgE level between the AST and ASS groups, and ACE treatment markedly reduced its expression (*P* < 0.01). In addition, we also detected the expression level of interferon-*γ* (IFN-*γ*), the principal Th1 cytokine. There was no obvious difference between the NC and NA groups. Compared with the NC and NA groups, the IFN-*γ* level significantly decreased in the AST and ASS groups (*P* < 0.01). There was no statistical difference between the AST and ASS groups, and the ACE treatment significantly increased the expression level of IFN-*γ* compared with the AST and ASS groups (*P* < 0.01).

Next, we used HE staining to observe the airway inflammation in each group of mice. As shown in Figure 3(b), in the NC and NA groups, smooth airway thickening and inflammatory cells infiltration were not observed. After OVA sensitization and stimulation, edema of airway epithelial cells and thicken of airway were observed. A large number of inflammatory cells around the airways were observed in both AST and ASS groups (*P*_AST,ASS_ < 0.01). After ACE treatment, although the inflammatory score was not statistically significant, visual observation suggested that these pathological changes had been markedly improved.

Since IL-13 can induce epithelial cells to secrete mucus in asthma [5], the airway mucus secretion in each group of mice was also observed. The PAS staining results (Figure 3(c)) showed that compared with the NC and NA groups, the percentage of positive staining of area of interest (AOI+ %) was significantly higher in the airway of the AST, ASS, and ASA groups (*P*_AST,ASS,ASA_ < 0.01). After ACE treatment, the percentage of positive staining in the AOI of the ASA group (*P*_ASA_ < 0.01) was significantly lower compared with the AST and ASS groups.

### 3.4. Acupoint Catgut-Embedding Downregulated Pulmonary ILC2s in OVA-Induced Asthmatic Mice

**Figure 4:**
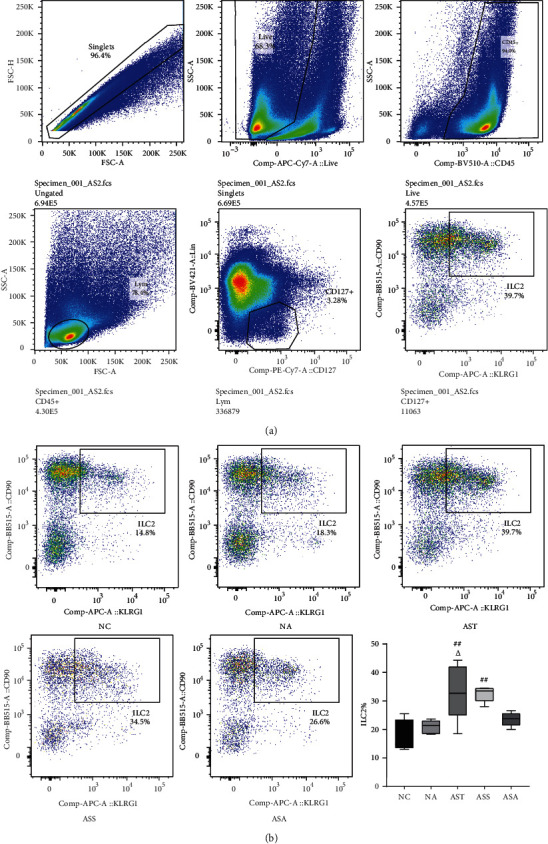
Acupoint catgut-embedding treatment downregulated the percentage of ILC2s in the lungs of asthmatic mice. (a) Gating process for ILC2s. (b) The percentage of ILC2s in each group. Data are presented as median (P25, P75), *n* = 5. ^##^*P* < 0.01 vs. NC group; *^Δ^P* < 0.05 vs. NA group. NC: control group; NA: control + acupoint catgut-embedding group; AST: asthma group; ASS: asthma + sham-acupoint catgut-embedding group; ASA: asthma + acupoint catgut-embedding group.

It has been shown that ILC2s produce the type 2 cytokines and are responsible for the initiation of the Th2 response [26], and the secretion of IL-4/IL-13 from Th2 cells also promotes the proliferation and expansion of the ILC2s population in the lungs [9]. Therefore, we detected the percentage of ILC2s in the lungs of mice in each group using flow cytometry. ILC2s were negative for lineage markers and positive for CD45, CD90.2, CD127, and killer cell lectin-like receptor G1 (KLRG1) [27–29]. As shown in Figure 4, there was no statistical difference in the proportion of ILC2s between the NC and NA groups, as well as between the AST and ASS groups. Compared with the NC and NA groups, the proportion of ILC2s in the lung tissues was significantly increased in the AST and ASS groups (*P*_AST,ASS_ < 0.01 vs. NC, *P*_AST_ < 0.05 vs. NA). After ACE treatment, the proportion of ILC2s in the ASA group showed a strong downward trend, which was consistent with the trend of IL-4 and IL-13 in other groups.

### 3.5. Acupoint Catgut-Embedding Inhibited the NF-*κ*B/COX-2 Pathway in Airways of OVA-Induced Asthmatic Mice

**Figure 5:**
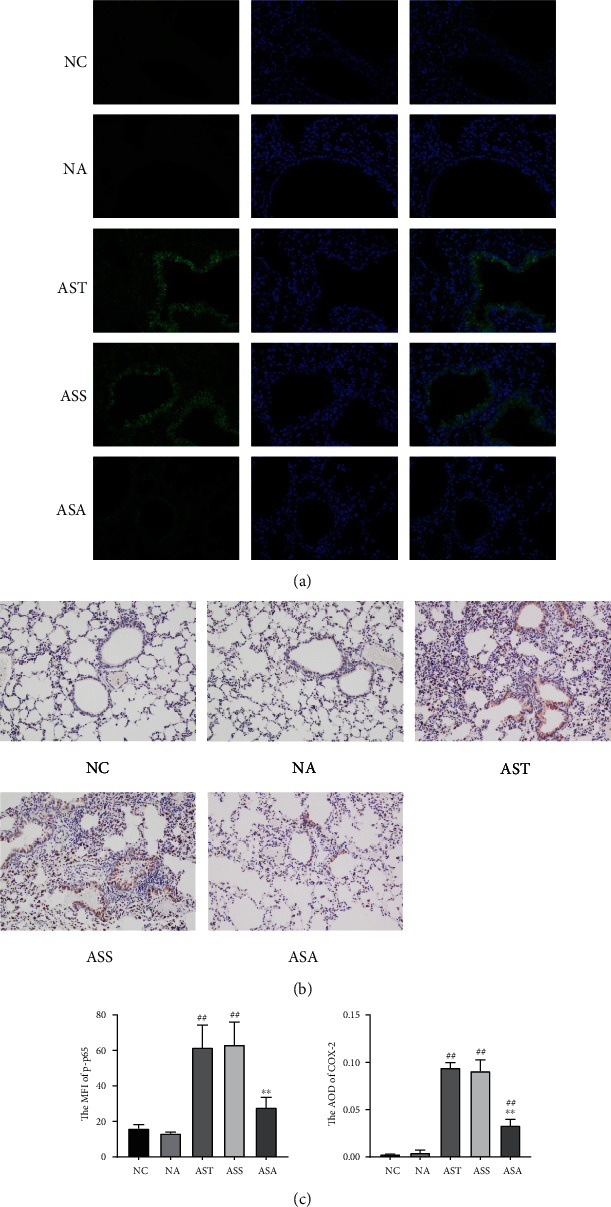
Acupoint catgut-embedding treatment inhibited the NF-*κ*B/COX-2 pathway in airways. (a) Immunofluorescence of p-p65 in each group. (b) Immunohistochemistry of COX-2 in each group. (c) The mean fluorescent intensity (MFI) of p-p65 and average optical density (AOD) of COX-2. Data are presented as mean ± SD, *n* = 5. ^##^*P* < 0.01 vs. NC and NA group; ^∗∗^*P* < 0.01 vs. AST and ASS group. NC: control group; NA: control + acupoint catgut-embedding group; AST: asthma group; ASS: asthma + sham-acupoint catgut-embedding group; ASA: asthma + acupoint catgut-embedding group.

Studies have shown that the NF-*κ*B/COX-2 pathway is necessary for controlling inflammation in the lungs and airways in allergic asthma [18]. In present study, we detected p-p65 and COX-2 using immunostaining methods. As shown in Figures 5(a) and 5(c), in the lung tissues of mice in the NC and NA groups, there was almost no positive expression of p-p65, while in the AST, ASS, and ASA groups, p-p65 was mainly expressed in the airway mucosa layer, and the MFI of p-p65 was significantly increased in the AST and ASS groups (*P*_AST,ASS_ < 0.01). There was no statistical difference between the AST and ASS groups. Compared with the AST and ASS groups, the MFI of p-p65 in the ASA group was significantly decreased (*P*_ASA_ < 0.01). Similarly, as shown in Figures 5(b) and 5(c), compared with the NC and NA groups, the AOD of COX-2 in the airways of AST, ASS, and ASA groups was significantly increased (*P*_AST,ASS,ASA_ < 0.01), and there was no statistical difference between the AST and ASS groups. Compared with the AST and ASS groups, the AOD of COX-2 in the ASA group was markedly decreased (*P*_ASA_ < 0.01), suggesting that ACE treatment might inhibit the expression of COX-2 via NF-*κ*B pathway in asthmatic mice.

## 4. Discussion

It is generally accepted that the Th2 cytokines such as IL-4 and IL-13 lead to allergic asthma phenotype, and studies have shown that OVA-induced asthma in mice is often accompanied by a significant increase in Th2 cytokine levels [30]. In present study, we observed the effects of ACE on allergic asthma and its underlying mechanism. Our results showed that after OVA induction, the mice had a significant increase in *R*_*L*_, while Cdyn was significantly decreased, suggesting the mice developed AHR. Resutls demonstrated by the classification and counting of inflammatory cells in BALF showed that eosinophils, neutrophils, lymphocytes, monocytes, and total leukocytes were significantly increased, and the levels of IL-4/13 in BALF were significantly increased. Furthermore, the deformation of airway epithelial cells, thickening of airway wall and alveolar septum, and inflammatory cell infiltration was observed in the lung tissues of OVA-induced mice, suggesting that the mouse model of allergic asthma was successfully established [31]. After ACE treatment, the lung function of asthmatic mice was effectively improved, and the levels of IL-4 and IL-13 in BALF were significantly reversed. Since IL-4 modulates the production of IgE, and IL-13 promotes airway mucus production [5, 25], we also observed the effects of ACE on the level of OVA-specific IgE in BALF and airway mucus production. As expected, both OVA-specific IgE in BALF and airway mucus production were markedly increased in asthmatic mice and significantly reduced after ACE treatment. Considering Th1 cells play an important role to downmodulate Th2 response in asthma, its main effector IFN-*γ* inhibits the production of IL-4 and reduces the number of eosinophils in BALF in asthma [32, 33]. Therefore, the expression level of IFN-*γ* was also detected, and the results showed that its expressions was decreased after OVA stimulation, which was consistent with other studies [34, 35], and increased significantly after ACE treatment. These results indicated that the ACE treatment was effective in regulation of the imbalance of Th1/Th2 and alleviated Th2-dominated inflammatory responses of asthmatic mice.

ILC2s have been newly identified to produce Th2 cytokines, and studies have shown that pulmonary ILC2s participate in the pathological process of allergic asthma [36, 37]. On one hand, ILC2s can release large amounts of IL-4, IL-5 and IL-13, thereby initiating and amplifying the type 2 immune response [7]. While on the other hand, Th2 cytokines such as IL-4/IL-13 can also promote the proliferation and expansion of pulmonary ILC2s in allergic asthma [9]. Therefore, we used flow cytometry to detect the proportion of pulmonary ILC2s of mice in each group. Consistent with studies above, our results showed that compared with the normal mice, the proportion of pulmonary ILC2s in asthmatic mice was significantly increased, and ACE treatment effectively reduced the proportion of pulmonary ILC2s, suggesting that ACE treatment inhibited the proliferation and expansion of Th2 cytokines and ILC2s in the lungs, and might inhibit their interactions, which eventually inhibited the Th2 inflammatory responses in allergic asthma.

Although IL-4/IL-13 were considered to play a role in allergic asthma mainly through the janus tyrosine kinases JAK1 and JAK3/signal transducer and activator of transcription-6 (STAT6) signaling, studies have shown that in addition to the JAK/STAT signaling, IL-4/IL-13 may also participate in the pathological process of asthma through other pathways such as NF-*κ*B signaling [12]. It is reported that IL-4 alone has no activating effect on NF-*κ*B; however, it can enhance the activation of NF-*κ*B induced by other factors such as IL-1 [10, 38]. As for IL-13, studies have shown that it induces the translocation of NF-*κ*B in human bronchial smooth muscle cells, and airway epithelium NF-*κ*B activation leads to a significant increase in IL-13 [11, 39]. The NF-*κ*B signaling pathway also plays a key role in regulating inflammation through COX transcription [40], as studies have shown that the COX-2 induced by NF-*κ*B is an important factor leading to the lung inflammation in allergic asthma [16, 18]. Therefore, we observed the effect of ACE treatment on the NF-*κ*B/COX-2 pathway by immunostaining. As expected, after OVA induction, the expression of p-p65 and COX-2 was significantly upregulated in lung tissues of asthmatic mice, and the ACE treatment remarkably inhibited their expression. However, the specific mechanism of the inhibitory effect of ACE treatment on NF-*κ*B/COX-2 pathway in lung tissues of allergic asthmatic mice is not clear, and it is difficult to determine whether this effect is direct or indirect. Further research is still required.

## 5. Conclusion

In conclusion, our study demonstrated that the ACE treatment alleviated AHR and inhibited Th2 responses and ILC2s in the lungs of OVA-induced allergic asthmatic mice, and the mechanism might be related to the inhibition of NF-*κ*B/COX-2 pathway.

## Data Availability

All underlying data is available on request through email address of corresponding authors.
